# Emergence of altruism behavior in army ant-based social evolutionary system

**DOI:** 10.1186/2193-1801-3-712

**Published:** 2014-12-04

**Authors:** Takumi Ichimura, Takuya Uemoto, Akira Hara, Kenneth J Mackin

**Affiliations:** Faculty of Management and Information Systems, Prefectural University of Hiroshima, 1-1-71, Ujina-Higashi, Minami-ku, 734-8558 Hiroshima, Japan; Graduate School of Information Sciences, Hiroshima City University, 3-4-1, Ozuka-higashi, AsaMinami-ku, 731-3194 Hiroshima, Japan; Department of Information Systems, Tokyo University of Information Sciences, 4-1, Onaridai, Wakaba-ku, 265-8501 Chiba, Japan

**Keywords:** Artificial life simulation system, Army ant, Altruism behavior

## Abstract

Army ants perform the altruism behavior that an ant sacrifices its own well-being for the benefit of another ants. They build bridges using their own bodies along the path from a food to the nest. We developed the army ant inspired social evolutionary system by using Swarm library. The system has 2 kinds of ant agents, ‘Major ant’ and ‘Minor ant’. They communicate with each other via pheromones. Army ant can recognize them as the signals from the other ants. The pheromones evaporate with the certain ratio and diffused into the space of neighbors stochastically. If the optimal bridge is found, the path through the bridge is the shortest route from the food to the nest. We define the probability for an ant to leave a bridge as to the number of neighboring ants. The constructing method of the optimal route has been proposed. In this paper, the behaviors of ant under the environment with two or more feeding spots were observed. Some experimental results show the behaviors of great interest with respect to altruism of ants. The knowledge discovery of social evolutionary process from some computer simulation results is described in this paper.

**Electronic supplementary material:**

The online version of this article (doi:10.1186/2193-1801-3-712) contains supplementary material, which is available to authorized users.

## Introduction

In animal societies, self-organization is the theory of how minimal complexity in the individual can generate greater complexity at the population. The rules specifying the interactions among the components in the system are implemented by using only local information without global information. In the study of social evolution, army ant performs altruism as one behavior of complexities, where each individual reduces its own fitness but increases the fitness of other individuals in the population. Such behaviors seem to be involved acts of self-sacrifice in order to aid the others. In evolutionary biology, such a behavior is called reciprocal altruism. The concept was initially developed to explain the evolution of cooperation as mutually altruistic acts (Trivers 1971). The basic idea is close to the strategy of “equivalent relation” in the study of strategic decision making.

Army ants are characterized by their two different phases of activities, a nomadic phase and a stationary phase. During the nomadic phase, army ants move during the day to capture insects, spiders, and so on. The stationary phase starts when the larvae pupate for a few weeks. Moreover, army ants build a living nest with their bodies instead of building a nest like other ants. Each ant will hold on to the other legs and form a linked chain or a ball structure. This behavior is known as a bivouac. This allows the bridging of an empty space. In order to address the self-assembled structure as a particular type of aggregation, Deneubourg *et al.* defined the probability of an ant entering or leaving chain in (Deneubourg et al. 2002). Moreover, they showed that the gregarious behavior facilitates cooperation by Blattella germanica in shelters during the resting period. The probability to leave the shelter was defined.

Ishiwata et al. (2011) developed the simulation system for the foraging behavior and the altruism of army ants by using Swarm library, *Swarm-2.2* (Lancaster et al. 2002). (The original website www.swarm.org is in the process of being rebuilt.) The probabilities to form the chain defined in (Lioni et al. 2001) was used in their simulation experiments. The number of neighboring active ants is considered as the condition for altruistic behavior. Their simulation results show a mimic altruistic behavior.

By inspiring Ishiwata’s study, Ichimura *et al.* developed the multi-agent simulation system to execute more realistic altruistic behavior where two or more kinds of agents realize the division of roles in army ants (Ichimura and Douzono 2012). According to the environment in (Ichimura and Douzono 2012), the simulation results reported that the optimal path from the food to the nest cannot be always found, because two or more chains in the environment were formed. Although more emergence of altruistic behaviors was observed, but the capabilities of forming chain was dispersed. As a result, the performance of foraging decreases and some ants took a circuitous route. On the contrary, Ichimura *et al.* defined the evaporation rate of pheromone dues to normal distribution probability and the probability to leave from the chain when the ants in its neighbor region depart gradually in (Ichimura and Douzono 2012). The altruism simulation results are reported to find more optimal paths from food to the nest.

In this paper, we observed the behaviors of ant agents under the multi feeding spots in the same environment of (Ichimura and Douzono 2012). Some experiments with different ratio of feed size were investigated. In general, ant agents take an action to be concentrated in the largest feeding spot. The shortest path from the spot to the nest is constructed and the ants bring feed to the nest. Then, the feeding spots will be disappeared in the order of larger spot. However, it has turned out that there is a certain tendency without regard to the size of feed. The altruism behavior does not work well and the bridge will be broken, if enough ant agents are not gathered into the ditch. As a result, the food at the spots remains to the end of simulation. Moreover, the group of agents was automatically constructed during search. The experimental results with the different number of agents show the building group for the effective search.

Such behaviors are also seen in the collaborative social networks. Especially, a research framework for studying social systems uses agent based modeling and simulation. Madey et al. (2002, 2003) describe the simulation results for the collaborative social network composed of open source software (OSS) developers and projects. The obtained knowledge in this paper will be useful for the collaborative social networks.

The remainder of this paper is organized as follows. The section Simulation environment describes the simulation environment with Swam library. The section Agent behaviors defines the behaviors of agents such as search phase, homing phase (return to the nest), and altruism phase. The section Proposed method describes the proposed method related to pheromone and the leaving probability from chain. Experimental results for simulations are described in the section Experimental results for altruism and the section Experimental results for the formation of group. In the section Conclusive discussion, we give some discussions to conclude this paper.

## Simulation environment

**Figure 1:**
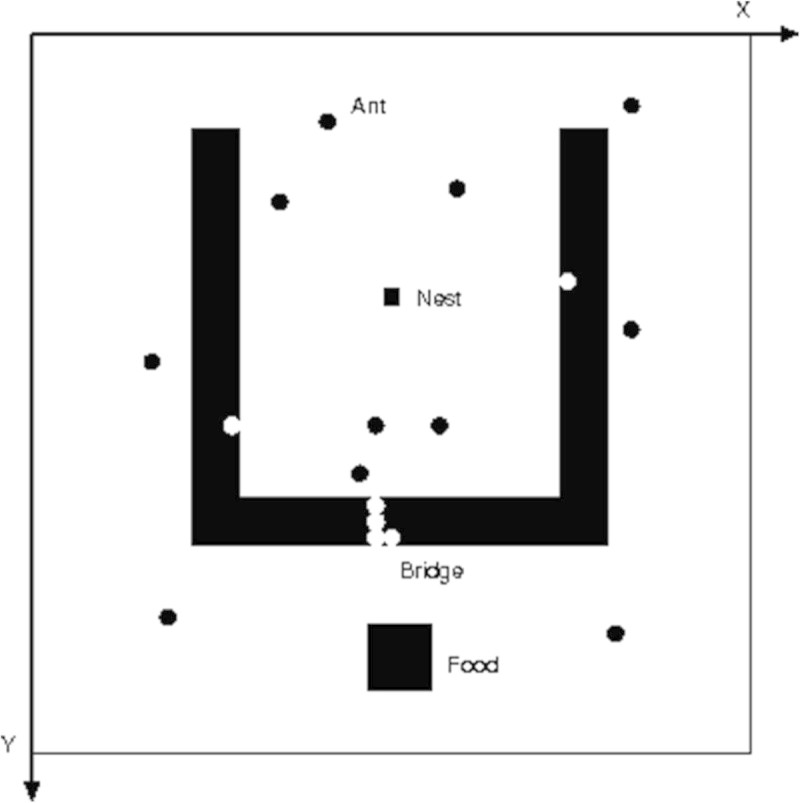
**Environment in army ant simulation system.**

The *swarm* is the basic unit of simulation for a collection of agents executing a schedule of actions. The *Swarm* provides object oriented libraries of reusable components for building models and analyzing, displaying, and controlling experiments on those models. We executed the altruism simulation system by pheromone evaporation and its diffusion in army ant multi agent systems. The developed system is depicted in 100×100 2D space as shown in Figure 1. The solid-filled rectangle, which consists of 3 kind of bars: ‘leftbar’, ‘rightbar’, and ‘centerbar’, divides the space into 2 parts. The inner part is the nest region and the bottom part under the rectangle is the food source part.

The 4 coordinates (x,y) of leftbar, rightbar, and centerbar are {(30, 30), (33, 30), (30, 70), and (33, 70)}, {(70, 30), (73, 30), (70, 70), and (73, 70)}, and {(30, 70), (73, 70), (30, 73), and (73, 73)}, respectively. Each bar represents a ditch and the width of ditch is 3. The center rectangle of the space is ‘nest’ and the bottom rectangle under the bar is ‘food source.’ Since an ant cannot cross the ditch by itself, some ants begin altruistic behavior to cooperate with each other. The two hypotheses were proposed as the judgment criteria for altruistic activity, Model 1: Based on the Presence of Neighboring Ants and Model 2: Based on the Presence of Pheromone (Ishiwata et al. 2011). In Model 1, an ant will start formation of living bridge over a gully only when neighboring ants are present. Hypothetically, this approach will be more efficient compared to forming a bridge blindly. In Model 2, the places where pheromone concentrations are higher than a fixed level are the locations that many ants have passed and/or will pass through in future.

**Figure 2:**
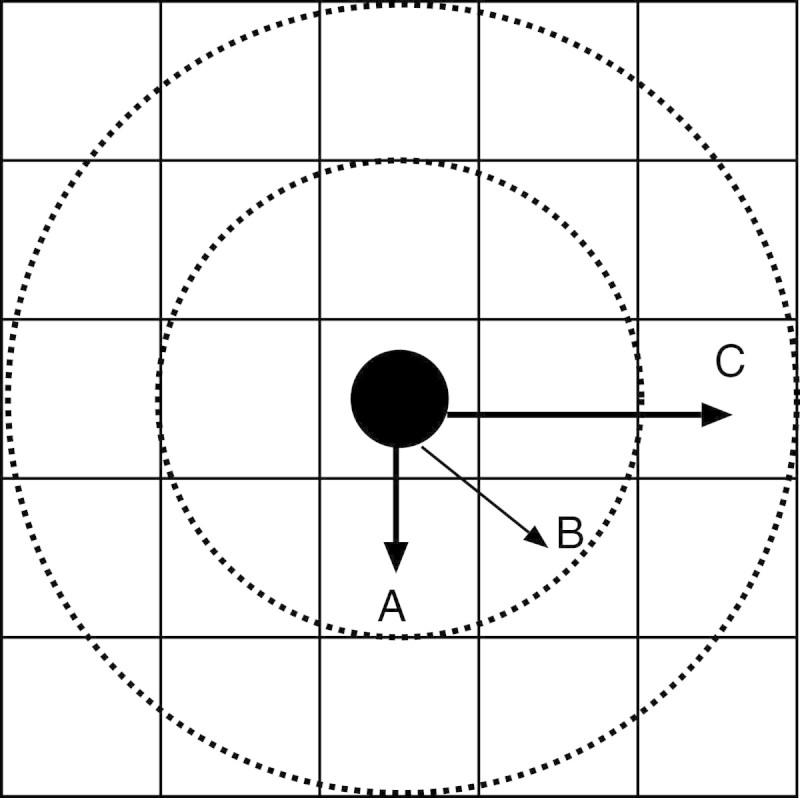
**The area of activities.**

Figure 2 shows the area of activities and the visual field by an army ant. In this paper, ant agents can move in the diagonal direction, but the scattered pheromone diminishes compared to the adjacent positions on up and down, left and right. The distance from a position to the neighbor is defined *Distance*. For example, the distance to ‘A’ and ‘C’ in Figure 2 are 1 *Distance* and 2 *Distance*, respectively. Practically,‘B’ is $\sqrt{2}\text{Distance}$. However, for the sake of ease, we define ‘B’ as 1 *Distance* in the diagonal direction.

## Agent behaviors

The actions include foraging for foods and transport of them and communications with neighboring ants using pheromone. The pheromone is released by an agent when it finds food. Once the pheromone is attenuated and is dispersed, the information about the food position is disseminated among the ants.

The system has 2 kinds of ant agents, ‘Major ant’ and ‘Minor ant’ and the ants communicate with each other via pheromones. Major ant scatters pheromones and moves throughout in the environment. On the other hand, Minor ant makes a mimic altruistic behavior to foraging and transporting. Ichimura *et al.* shows the numerical superiority in case of the 2 variants of ants (Ichimura and Douzono 2012). In this paper, the experimental simulation related to the altruistic behaviors has the 2 kinds of ant agents.

A minor ant agent aims to find a food source and then to return to the nest. If there is a ditch in the path among them, the ant will build a bridge. The 3 kind of states are defined according to the behavior of ants (Ishiwata et al. 2011).

**Search state**

Search state is an initial condition of agents to seek the food source by random walk. Once an agent reaches the food, it moves into Return state. The ant takes a food on the way back to the nest until the food source becomes empty. Below shows the search algorithm.

1. Search of food secure
  First, search a food source within 1 *Distance*. If the agent finds a good at the destination, moving into Return state.
2. Perception of pheromone
  Perceiving pheromone within 2 *Distances*.
3. Search the other agents
  Checking the other agents within 2 *Distances*. If the other agents stay, go to 4). Otherwise go to 6).
4. Search a ditch
  Search a ditch within 1 *Distance*. If there is a ditch, transit to Altruism State. otherwise go to 6).
5. Move
  Move to the other position according to the scattered pheromone described in the section “Pheromone update”.
6. Random selection of walking direction
  Check whether the other agent stays or a ditch exists at the next position except going straight ahead. The next position is selected with an arbitrary probability *α*. If the position is empty, go back to 1). Otherwise, select another position. Moreover, if the agent is surrounded by other agents or a ditch. it stays at the same position until the neighbor becomes empty.

**Return state**

In Return state, an agent comes back to the nest carrying the food. After reaching the nest, the agent moves into Search state. Below shows the return algorithm.

1. Current position
  Check the current position of an agent. It is in the nest, go to Search state. Otherwise, it goes to next step to move to the nest.
2. Search a ditch
  If there is a ditch within 1 *Distance*, go to 3). Otherwise, it moves a next position to the next and go to 1).
3. Random walk
  Check wether the the other agent stays or ditch exists at the randomly selected next position except going straight ahead. If the position is empty, go back to 1). Otherwise, select another position. Moreover, if the agent is surrounded by other agents or a ditch, it stays at the same position until the neighbor becomes empty.

**Altruism state**

Some agents stop walking before a ditch and come together asflock. Two or more agents will build a bridge. below shows the altruism algorithm.

1. Search the other agent
  if there are *n* agents within 2 *Distances*, the agent stays with an arbitrary probability 1 - *P*_1_ described in the section “The model of army ant” and continues to check its surrounded situation. Otherwise, go to 2) with the probability *P*_1_.
2. Go to search state

Select a position within 1 *Distance* in the part of chain. If the position is empty, go to position, and then make the transition to Search state. Otherwise, the ant is embedded in the chain.

### Pheromone update

In many works related ant systems, the ants communicate with each other via the pheromone dissemination. However, the researchers have discussed only about the pheromone on the ground. We consider that the pheromone evaporates and spreads into the space. The ant in this study can recognize the volatilization of pheromone in the space, but not know the pheromone on the ground. Based on such an idea, pheromone update process is executed by Eq. (1) and Eq. (3).

$$\begin{array}{l} \text{spac}e_{\left(x,y\right)}^{\prime }(t)=r_{A}\times {\text{space}}_{\left(x,y\right)}(t)\\ \;+r_{B}\times \;\left(\underset{p}{\sum }{\text{space}}_{\left(i_{p},j_{p}\right)}(t)-4{\text{space}}_{\left(x,y\right)}(t)\right)\\ \;+r_{C}\times \;\left(\underset{q}{\sum }{\text{space}}_{\left(i_{q},j_{q}\right)}(t)-4{\text{space}}_{\left(x,y\right)}(t)\right) \end{array}$$

1 $$\begin{array}{lll} \left(x_{i_{p}},y_{j_{p}}\right) & = & \left\{(x,y+1),(x,y-1),(x+1,y),(x-1,y)\right\}\\ \left(x_{i_{q}},y_{j_{q}}\right) & = & \{(x+1,y+1),(x+1,y-1),\\ \left(x-1,y+1),(x-1,y-1)\right\} \end{array}$$

2 $$\begin{array}{l} {\text{space}}_{\left(x,y\right)}(t+1)=\text{spac}e_{\left(x,y\right)}^{\prime }(t)+r_{e}*{\text{ground}}_{x,y}(t) \end{array}$$

3 $$\begin{array}{l} \;{\text{ground}}_{\left(x,y\right)}(t\;+\;1)={\text{ground}}_{\left(x,y\right)}(t)\;-\;r_{e}*{\text{ground}}_{\left(x,y\right)}(t) \end{array}$$

where *space*_(*x*,*y*)_ means the amount of pheromone in the space over the position (*x*,*y*) in Eq. (1). *r*_*A*_ is the decay ratio. *r*_*B*_ is the diffusion rate in the direction of up and down, left and right. *r*_*C*_ is the diffusion rate in the direction of the diagonal. *ground*_*x*,*y*_(*t*) means the pheromone amount on the ground at the position (*x*,*y*) in Eq. (2). *r*_*e*_ is the evaporation rate.

### Multi feeding spots

**Figure 3:**
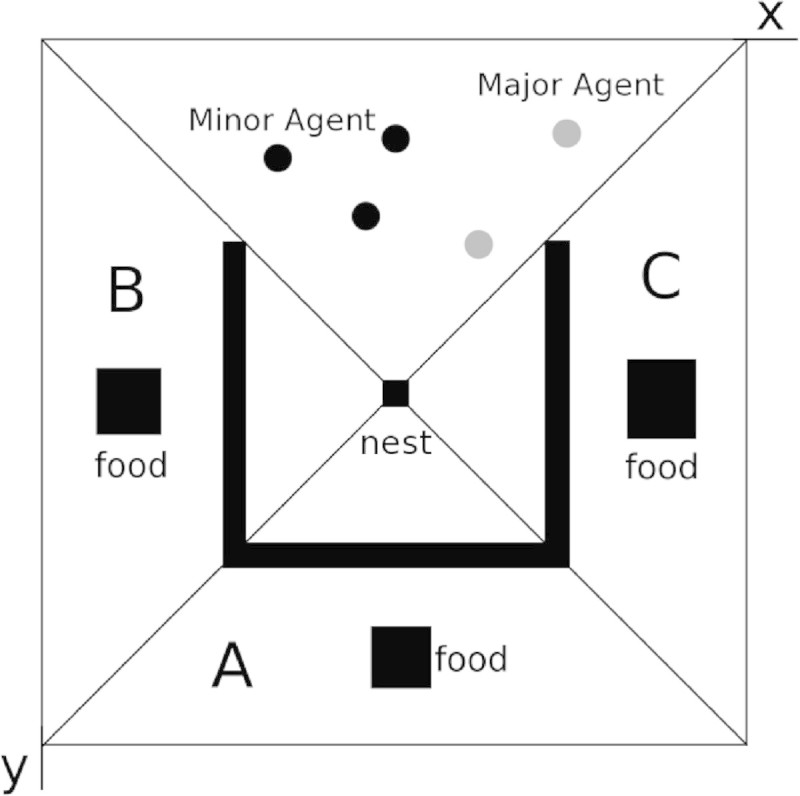
**The environment for multi feeding spots.**

Figure 3 shows the environment with multi feeding spots to extend the simulation system. As shown in Table 1, we investigate the behaviors of ant for some ratios of food size.

**Table 1:** **The size of feeding spots**

Env.	Agents	A:B:C	Env.	Agents	A:B:C
1-1		2:1:1	2-1		2:1:1
1-2	Major:3	1:2:1	2-2	Major:3	1:2:1
1-3	Minor:100	1:1:2	2-3	Minor:50	1:1:2
1-4		4:2:1	2-4		4:2:1

## Proposed method

The simulation system mainly focuses two parts, ‘Pheromone Evaporation and Its Diffusion’ and ‘Probability for leaving from chain’. In this paper, the pheromone evaporation method and a new probability for leaving from chain are defined as follows.

### Pheromone evaporation and its diffusion

As for the former part, Pheromone Evaporation and Its Diffusion, the method in (Ichimura and Douzono 2012) assumed the improper rate of evaporation and diffusion of pheromone in the agent and its behavior. The parameter setting causes a bias for the flock. That is, there is much pheromone in simulation environment partially. The situation increases the agents swarming around them. As a result, it becomes easy to enter Altruism State and two or more bridges are built without the shortest path from a food source to the nest.

**Figure 4:**
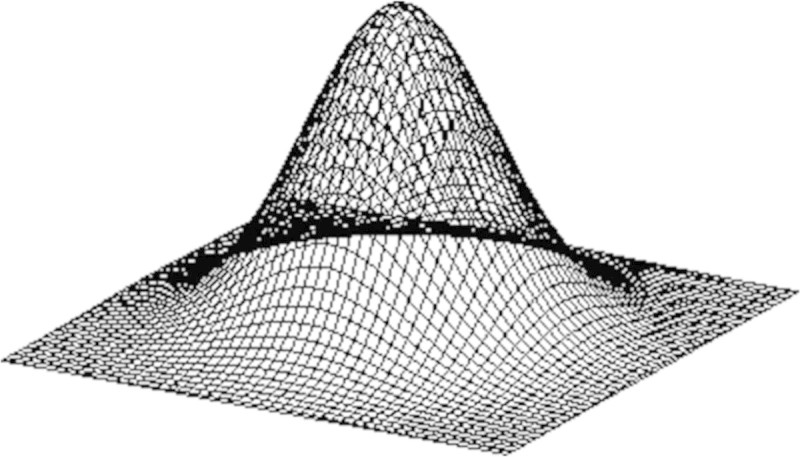
**The distribution of pheromone.**

**Figure 5:**
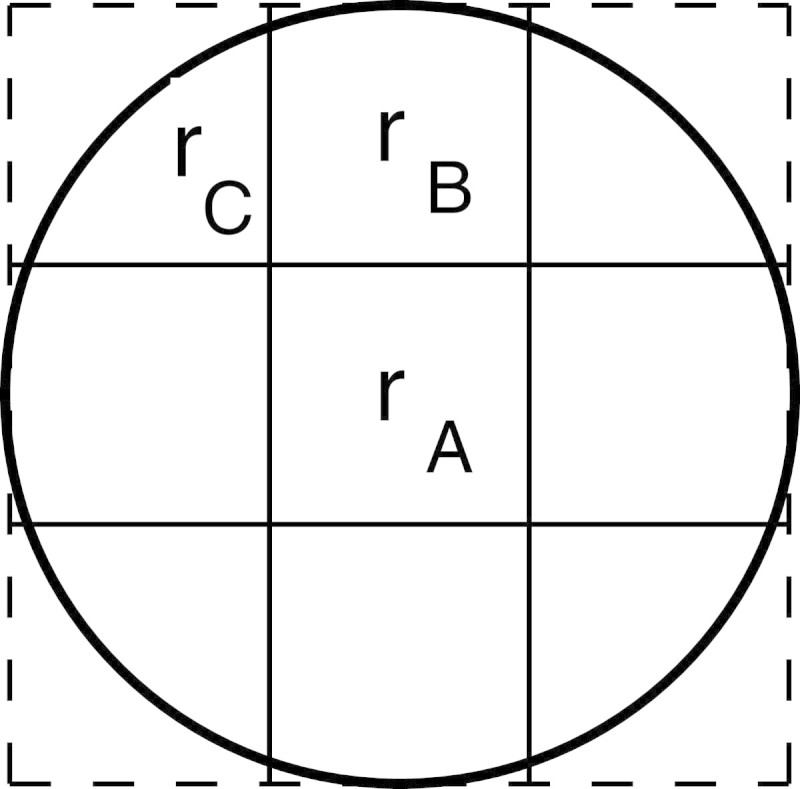
**The evaporation rate of pheromone.**

In order to avoid such a situation, the ratio of pheromone evaporation is defined based on the normal distribution probability as shown in Figure 4. Figure 5 shows the transverse plane of Figure 4. The rate of pheromone in 3D space is set to ‘ *r*_*A*_’: ‘ *r*_*B*_’: ‘ *r*_*C*_’ = 0.788:0.043:0.010 in Figure 5.

### The model of army ant

The probabilities of an ant entering a chain(*P*_*e*_) or leaving a chain (*P*_*l*_) are depending on the size of the chain. The chain is a small part of constructing bridge. Lioni et al. (2001) defined these probabilities as follows.

4 $$\begin{array}{l} P_{e}=C_{e0}+\frac{C_{e1}X_{i}}{C_{e2}+X_{i}} \end{array}$$

5 $$\begin{array}{l} P_{l}=C_{s0}+\frac{C_{s1}}{C_{s2}+X_{i}^{\nu }} \end{array}$$

where *X*_*i*_ is the number of ants in the chain *i*. *C*_*e* 0_, *C*_*e* 1_, and *C*_*e* 2_ are parameters for entering the chain. *C*_*s* 0_, *C*_*s* 1_, and *C*_*s* 2_ are parameters for leaving the chain. The size of the agent group when they leave a chain will be larger than that of the initial group. *ν* is a parameter of the growing rate of group during the construction of chain. The values of these probabilities should be in the range of 0 and 1.

The function *P*_*e*_ expresses the idea that the probability for an ant to join the chain grows the number of nest mates already presented and reaches a plateau value equal to *C*_*e* 0_+*C*_*e* 1_. *C*_*e* 0_ is the value of spontaneous hanging when *X*_*i*_=0. The function *P*_*l*_ expresses the probability for an ant to leave the chain decreases with *X*_*i*_.

The ant in the chain does not always stay in the same chain. A certain probability for leaving from the chain is required to realize Altruism Status. Due to interaction between ants, the probability decreases with the number of con-specifics in the chain. The phenomena is ruled by empirical equation very similar to that for Oecophylla (Deneubourg et al. 2002). The probability for leaving from chain is given by Eq. (6).

6 $$\begin{array}{c} P_{i}=\frac{a}{1+bX_{i}^{2}} \end{array}$$

where *X*_*i*_ is the number of ants in the chain *i*. *a* is the probability of leaving a chain under a disregard for the number of other agents. *b* is the parameter of depending the amount of pheromone in chain *i*: *b*= min{*η*(log(*space*_(*x*,*y*)_+1))+*ε*,1}. A theoretical model suggests that these basic mechanisms account for the clustering of insects (Deneubourg et al. 2002; Lioni and Deneubourg 2004; Lioni et al. 2001).

**Figure 6:**
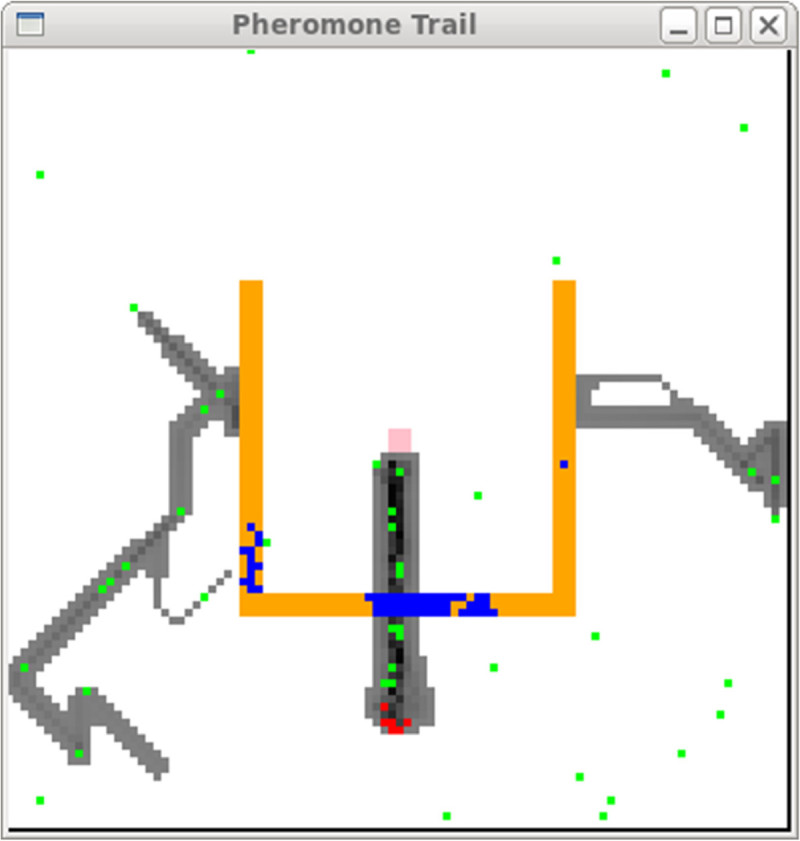
**The discover of shortest path.**

Ichimura et al. (2012) reports the agents in altruism situation perform the shortest path by construction of the bridge. Figure 6 shows the constructed bridge on the way from the feeding spot to the nest. In this paper, we investigate the altruism situation by using the condition in the section **Altruism State** for entering a chain and Eq. (6) for leaving the chain.

## Experimental results for altruism

The behavior of army ants at each environment as shown in Table 1 was observed. Parameter settings are as follows: *n*=2, *a*=0.4, and *r*_*e*_=0.05. At each environment in this paper, 10 trials for each set were executed and the behavior of ants were recorded as the motion video. There are 2 kinds of ants at each environment, Major ants to make a random search and Minor ants to follow the scattered pheromone. In this paper, for almost trials we can observe the following simulation results. During the initial phase as the search of an area for prey, the movement of Major agent will be a key in the change of course to search the subspace, because the Major agent scatters the pheromone while moving in a space. After the discovery of food, the Minor agent catches the food and scatters the pheromone on the way from the spot to the nest. The path becomes congested since there is an obstacle of a ditch. Such situation causes the construction of the bridge on the ditch, since the ants search the shortest path from the feeding spot to the nest. Moreover, the agent swarming around the food increases with the size of food, because more pheromone is scattered while the agent brings a food to the nest. That is, the larger the food in the environment has, the more pheromone the agents scattered.

### Transition of 100 agents

**Figure 7:**
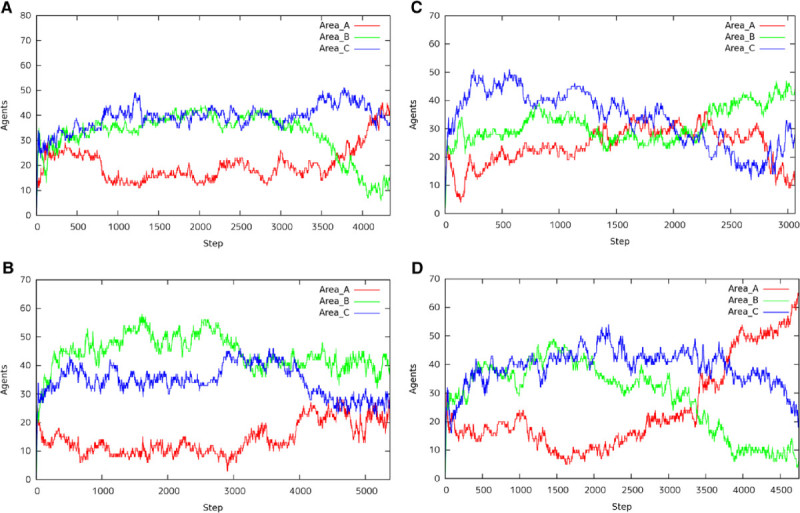
**Transition of 100 Agents.** **(A)** A:B:C=2:1:1, **(B)** A:B:C = 1:2:1, **(C)** A:B:C = 1:1:2 and **(D)** A:B:C = 4:2:1.

Figures 7 show the number of agents in each area, A, B, and C, with 100 agents in the environment. In case of 100 agents, they are divided into some groups and each group can search the area, respectively. Figure 7(A), Figure 7(B), Figure 7(C), and Figure 7(D) show the transition of agents with *A*:*B*:*C*={2:1:1, 1:2:1, 1:1:2, 4:2:1}, respectively. The transition of agents as shown in these figures emerged 7 or 8 times of 10 trials in each situation. We can observe that ant agents carry the food from the nearest food spot or the largest size of food spot. Note that even if the food size of area *A* is the largest, the food of area *A* remains until area *B* or area *C* is disappeared. Because ant agents find areas *B* or *C* and bring all food from area *B* or area *C*, respectively.

**Figure 8:**
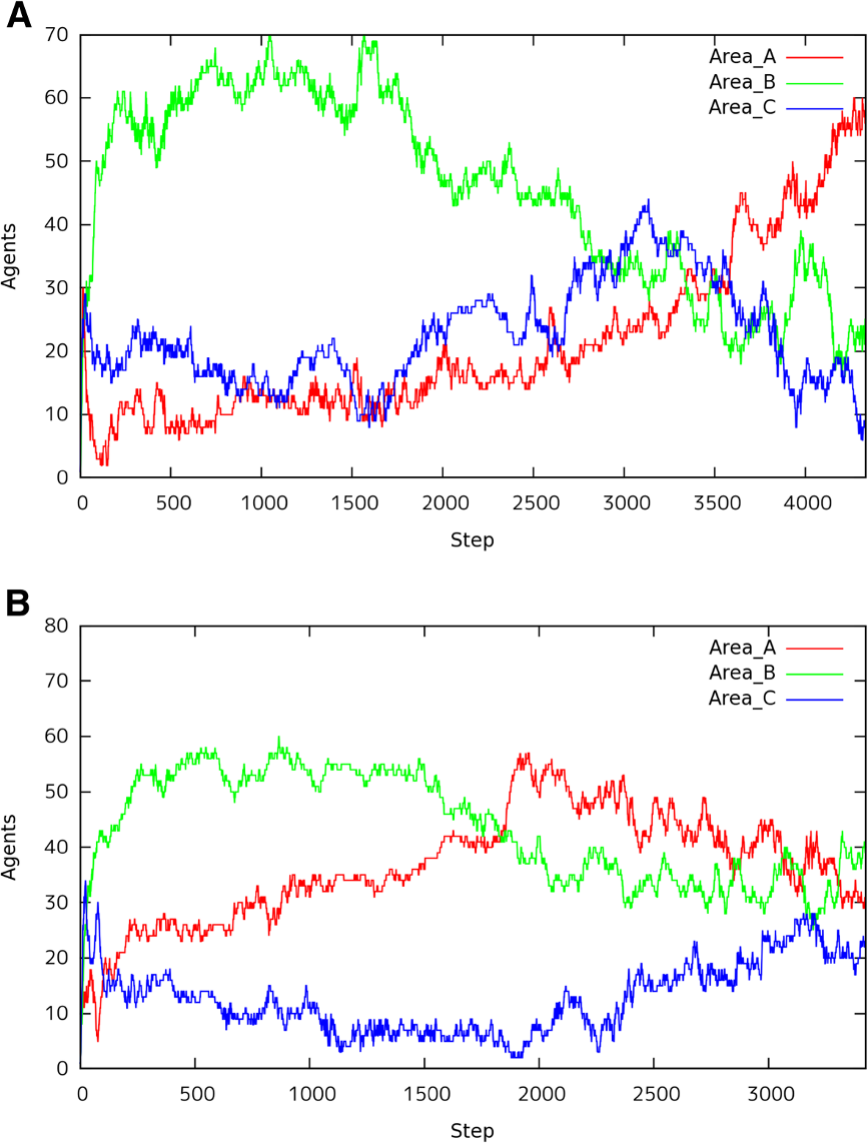
**Transition of 100 Agents (other case).** **(A)** A:B:C = 2:1:1 and **(B)** A:B:C = 4:2:1.

On the contrary, Figure 7(C) (*A*:*B*:*C*=1:1:2), Figure 8(A) (*A*:*B*:*C*=2:1:1), and Figure 8(B) show that the results are beyond our expectations. In the experiment as to Figure 7(C), more agents gathered to the areas *B* and *C* than the area *A* at the initial phase. And then agents moved to area *C* larger than area *B* and they made a bridge in the area *C*. After the food was lost in area *C*, the ants in area *C* moved to area *A*. We expected area *B* than area *A* will be disappeared, because some agents visited area *B* and they knew the existence of area *B*. However, most agents moved to *A*, because the pheromone at the initial phase was disappeared. Therefore, the food of *B* was carried to the nest after area *A* was lost.

In the experiment as to Figure 8(A), agents gathered to area *B* too much. As a result, the wide bridge was constructed between area *B* and the nest. Because almost agents were in altruism state and only a few agents could carry the food to the nest, it took a long time to finish the work in area *B* and they could not proceed to the area *A*. Some agents in area *B* moved to area *C* according to pheromone scattered by Major ants. After the agents finished the work in area *C*, they could move to area *A* at last. When ant agents concentrated superfluously, the search might not progress.

In the experiment as to Figure 8(B), agents gathered to area *B* and the bridge between area *B* and the nest was constructed. Some agents in area *B* were attracted by the largest size of area *A*. As a result, because there were not almost agents in area *C*, the bridge was not constructed in area *C* and the agents avoided the ditch to carry the food.

We summary the characteristic behaviors under the 100 agents as follows.

1. Most agents gather to the left area and the right area near from the nest. They move to the bottom area after they finish the work at each area.
2. Pheromone scattered by Major agents attracts the minor agents and they move to one of the left area and the right area. They move to the bottom area after they finished the work at the area.
3. Some agents make a bridge on the way from the nest to the food spot.

### Transition of 50 agents

**Figure 9:**
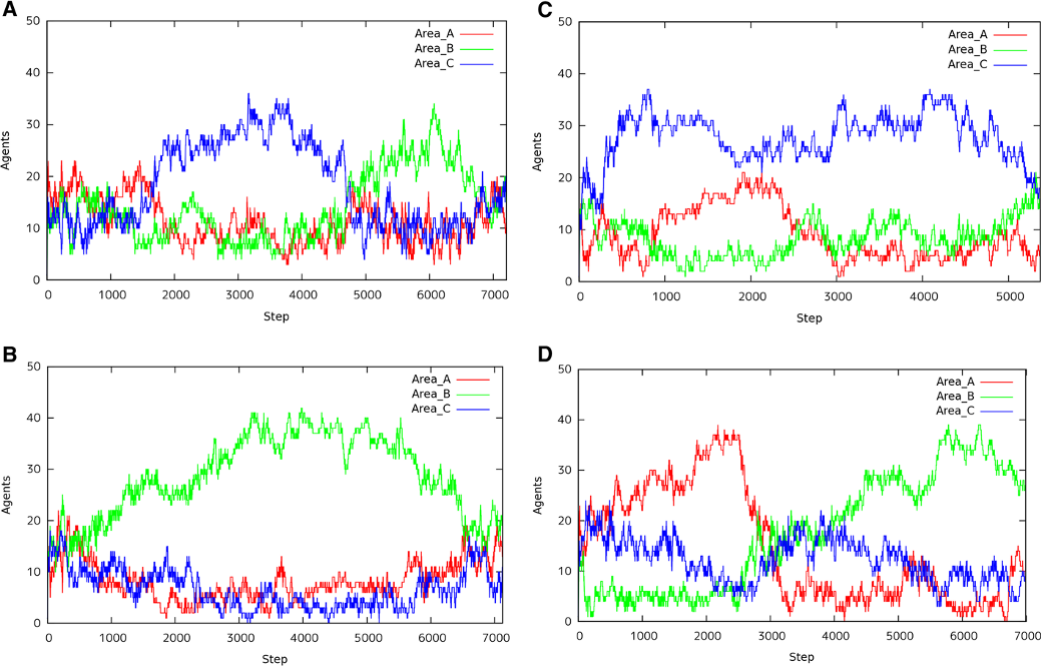
**Transition of 50 Agents.** **(A)** A:B:C = 2:1:1, **(B)** A:B:C = 1:2:1, **(C)** A:B:C = 1:1:2, and **(D)** A:B:C = 4:2:1.

Figures 9 show the number of agents in each area, A, B, and C, with 50 agents in the environment. The situation has 50 agents in the environment, they cannot search the space sufficiently. The ants did not divide into some search group and then the search of area was processed sequentially such as *A*→*B*→*C*. Figure 9(A) and Figure 9(D) show the transition of agents in case of *A*:*B*:*C*={2:1:1,4:2:1}, respectively. As shown in these figures, we can observe general behaviors in the search space. That is, the feeding spots are disappeared in the order of larger spot. On the contrary, Figure 9(B) (*A*:*B*:*C*=1:2:1) and Figure 9(C) (*A*:*B*:*C*=1:1:2) show that the result is beyond our expectations. In case of Figure 9(B), the constructed bridges were not in the shortest path on the way to the nest as shown in Figure 8(B). In case of Figure 9(B), it is an interesting case, and almost agents will make bridges place to place in the ditch from area *C* to the nest. The remaining agents should deliver the food to the nest, however very few agents cannot take all of them. The scattered pheromone was smaller than the evaporated pheromone. Therefore, the pheromone around the bridge disappears and then the bridge was destroyed, because the agents in altruism situation depart the bridge in the condition of less pheromone. The agent leaving from the bridge moves to area *A*, but there are only a few agents in the area. From such results, only few agents in the environment with large size of food cannot get the altruism situation easily.

We summary the characteristic behaviors under the 50 agents as follows.

1. Most agents gather to the left area and the right area near from the nest. They move to the bottom area after they finish the work at each area.
2. If the food size is the largest in the bottom area, the agents gather there regardless of the distance from the food spot to the nest. They separate to move the remaining spot after they finish the work at the area (See Additional file 1).

## Experimental results for the formation of group

The group of agent was automatically constructed during search. The experimental results with the different number of agents show the formation of group for the effective search. In this paper, the transition of {30,40,50,60,80,100,120} agents in the environment were investigated at 10 trials.

**Figure 10:**
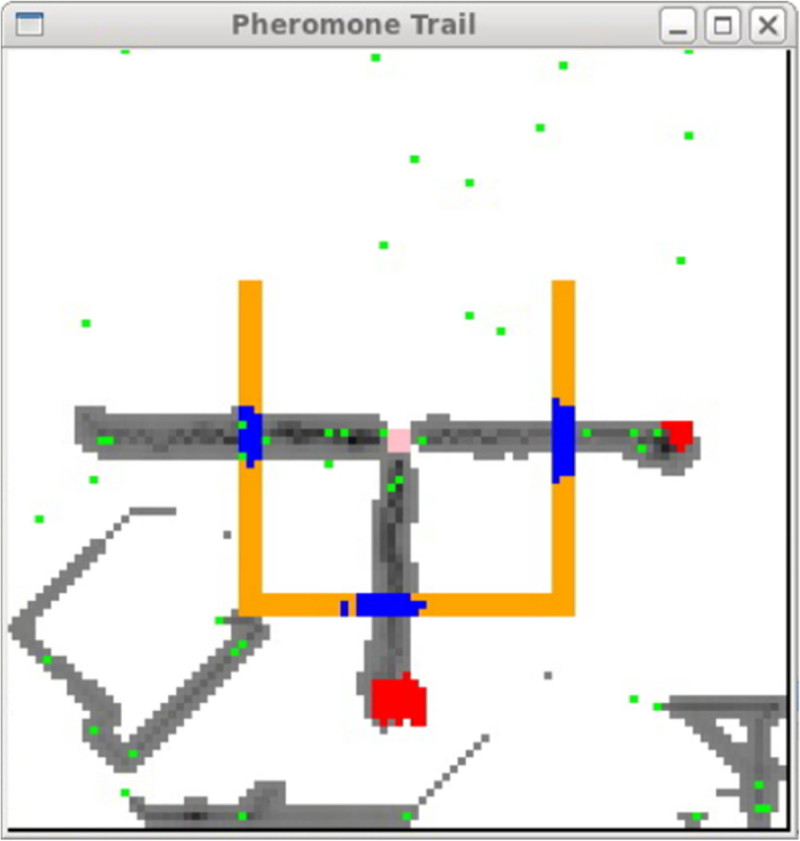
**The construction of 3 bridges simultaneously.**

In case of 30 agents, the narrow bridge appeared but was not the shortest path. The agent departs from the bridge and makes a bridge at a new place during the search if the bridge constructed at initial phase is not shortest path, and then they can find the optimal path from the food to the nest. In case of 50 agents, the group did not appear at any time. In case of 60 agents, however, agents are divided into two groups in 8 trials. The search by one group was observed only in 2 trials. In cases of 80 agents and 100 agents, the agents are always divided into 2 groups (See Additional file 2). In case of 120 agents, 2 groups or 3 groups were performed. For the case of 3 groups, there were the shortest path from each food spot as shown in Figure 10. The ants can carry all the food in the shortest time. Table 2 shows the relation between the group and agent. *N*_*Agent*_, *N*_*Group*_, *N*_*AgentOfGroup*_, and *N*_*Others*_ are the number of agents, the number of performed groups, the average number of agents in the group, and the number of nonparticipating agents to group.

**Table 2:** **The number of agents and the group**

***N***_***Agent***_	***N***_***Group***_	***N***_***AgentOfGroup***_	***N***_***Others***_
30	0	-	-
40	1	25	15
50	1	30	20
60	1	35	25
	2	25	10
80	2	30	20
100	2	40	20
120	2	50	20
	3	35	15

## Conclusive discussion

We developed the army ant inspired social evolutionary system which can perform the altruism. There are 2 kinds of ant agents communicated with each other via pheromones. Moreover, the pheromones evaporate with the certain ratio and diffused into the space of neighbors stochastically. In order to avoid the over-concentration in the chain, the probability of leaving from a chain is introduced. The system with the facilities can find the optimal place of bridge. The path through the bridge is the shortest from foods to the nest. In this paper, the behaviors of ant under the environment with multi feeding spots and the adequate number of agents were observed. The altruism behavior in the few agents to the size of food spot is hard to keep its situation. Such observations of behaviors in the computer simulation strongly will contribute to the shift to knowledge and power from the individual to the collective. We will explore how agent-based modeling and simulation can be used as a research technique to study collaborative social networks. The altruism behaviors described in the paper will be useful to discover the power of the synergy effect in social networks.

## Electronic supplementary material

Additional file 1:**Movie for 50 agents a:b:c** ***=*** **4:2:1.**(FLV 14 MB)

Additional file 2: Movie for 100 agents a:b:c *=* 4:2:1.(FLV 8 MB)
